# Is alcohol and psychoactive medication use associated with excess hospital length-of-stay and admission frequency? A cross-sectional, observational study

**DOI:** 10.1186/s12873-024-00979-y

**Published:** 2024-04-16

**Authors:** Danil Gamboa, Saranda Kabashi, Benedicte Jørgenrud, Anners Lerdal, Gudmund Nordby, Stig Tore Bogstrand

**Affiliations:** 1 Department of Medicine, Lovisenberg Diaconal Hospital, Oslo, Norway; 2 Department of Forensic Sciences, Oslo University Hospital, Oslo, Norway; 3 Institute of Health and Society, Faculty of Medicine, University of Oslo, Oslo, Norway; 4 Research Department, Lovisenberg Diaconal Hospital, Oslo, Norway

**Keywords:** Psychoactive medication, Alcohol, Length-of-stay, Admission frequency

## Abstract

**Background:**

Hospital length-of-stay and admission frequency are commonly used indicators of disease burden and health resource expenditures. However, the impact of psychoactive prescription medication use and harmful alcohol consumption on both the duration and frequency of hospital admissions is under-explored.

**Methods:**

We conducted an analysis of data gathered from 2872 patients admitted to the Emergency Department at Lovisenberg Diaconal Hospital in Oslo, Norway. Psychoactive medicines (benzodiazepines, opioids, and z-hypnotics) were detected via liquid chromatography-mass spectrometry analysis of whole blood, while alcohol consumption was self-reported through the Alcohol Use Disorder Identification Test-4 (AUDIT-4). Using logistic regression, we examined associations with our primary outcomes, which were excess length-of-stay and admission frequency, defined as exceeding the sample median of 3.0 days and 0.2 admissions per year, respectively.

**Results:**

Compared to the absence of psychoactive medication, and after adjusting for age, gender, malignant disease, pre-existing substance use disorder and admission due to intoxication, the detection of two or more psychoactive medicines was associated with both excess length-of-stay (odds ratio [OR], 1.60; 95% confidence interval [CI], 1.20 to 2.14) and yearly hospitalization rate (OR, 3.72; 95% CI, 2.64 to 5.23). This association persisted when increasing the definition for excess length-of-stay to 4 and 5 days and to 1.0 and 1.5 admissions per year for admission frequency. Harmful alcohol consumption (AUDIT-4 scores of 9 to 16) was not associated with excess length-of-stay, but with excess admission frequency when defined as more than 1.0 admission per year when compared to scores of 4 to 6 (OR, 2.68; 95% CI, 1.58 to 4.57).

**Conclusions:**

Psychoactive medication use is associated with both excess length-of-stay and increased antecedent admission frequency, while harmful alcohol consumption may be associated with the latter. The utility of our findings as a causal factor should be explored through intervention-based study designs.

**Supplementary Information:**

The online version contains supplementary material available at 10.1186/s12873-024-00979-y.

## Background

Hospital length-of-stay and admission frequency are ubiquitous metrics in biomedical research, functioning as indicators for disease burden and health care expenditures [1–5].There is considerable interest in identifying factors related to either prolonged or frequent hospitalization [6, 7], with subsequent implementation in predictive models and risk stratification tools. Although several dimensions have been assessed, the utility of attributes other than age and co-morbidity ranges between contextual and ambiguous [8, 9]. Furthermore, while alcohol consumption and the use of psychoactive prescription medication is frequently encountered in clinical practice, data regarding their interaction with length-of-stay and admission frequency is sparse. For instance, the impact of alcohol use has been considered within the wider context of various substance use disorders in relation to hospitalization frequency [10], discounting lower, but still potentially harmful degrees of drinking [11]. Studies examining associations between alcohol consumption and length-of-stay appear to be limited to instances of intoxication [12] or in specialized subsets of patients [13]. Similarly, the role of benzodiazepines, opioids and z-hypnotics has generally been examined as part of a much larger and comprehensive list of potentially inappropriate medication among older, frequently admitted adults [14].

The above limitations are made more conspicuous by the considerable onus drinking and psychoactive medication use exerts on health care resources - alcohol use was related to 9.5% of all Emergency Department (ED) presentations in a multi-center study [15]. In the United States, adverse events attributable to benzodiazepine use accounted for an estimated 212,770 ED-visits annually [16], while since 2007, more than 10 000 overdose deaths each year have been attributed to prescription opioids [17]. Concurrent use of medication from different psychoactive drug classes appears to be prevalent, with a large proportion of prescription opioid users also being prescribed a benzodiazepine [18]. In Norway, the overall 1-year prevalence of z-hypnotic use was 9%, with co-medication with benzodiazepines and opioids being more frequent among younger long-term users [19]. In the community-dwelling elderly, usage rates for benzodiazepines, opioids, and z-hypnotics were 12.0%, 12.4% and 19.0%, respectively [20].

Psychoactive medication use appears to be more common in the hospitalized elderly when compared to population level-data [21], while smaller prevalence studies [22] suggest that many instances of alcohol use remains undetected among admitted patients, irrespective of age. In recent data published by our research group, 32.3% of more than 2600 blood samples drawn from acutely hospitalized adult patients were positive for at least one z-hypnotic, benzodiazepine or prescription opioid [23], while 21.1% of patients self-reported either hazardous or risky drinking, as measured by AUDIT-4 [24]. With usage rates available and employing post-discharge data, we therefore sought to investigate the relationship between the degree of psychoactive medication and alcohol use, and excess length-of-stay and frequent hospitalization among acutely admitted Internal Medicine-patients.

## Methods

### Study design, setting and participants

**Fig. 1:**
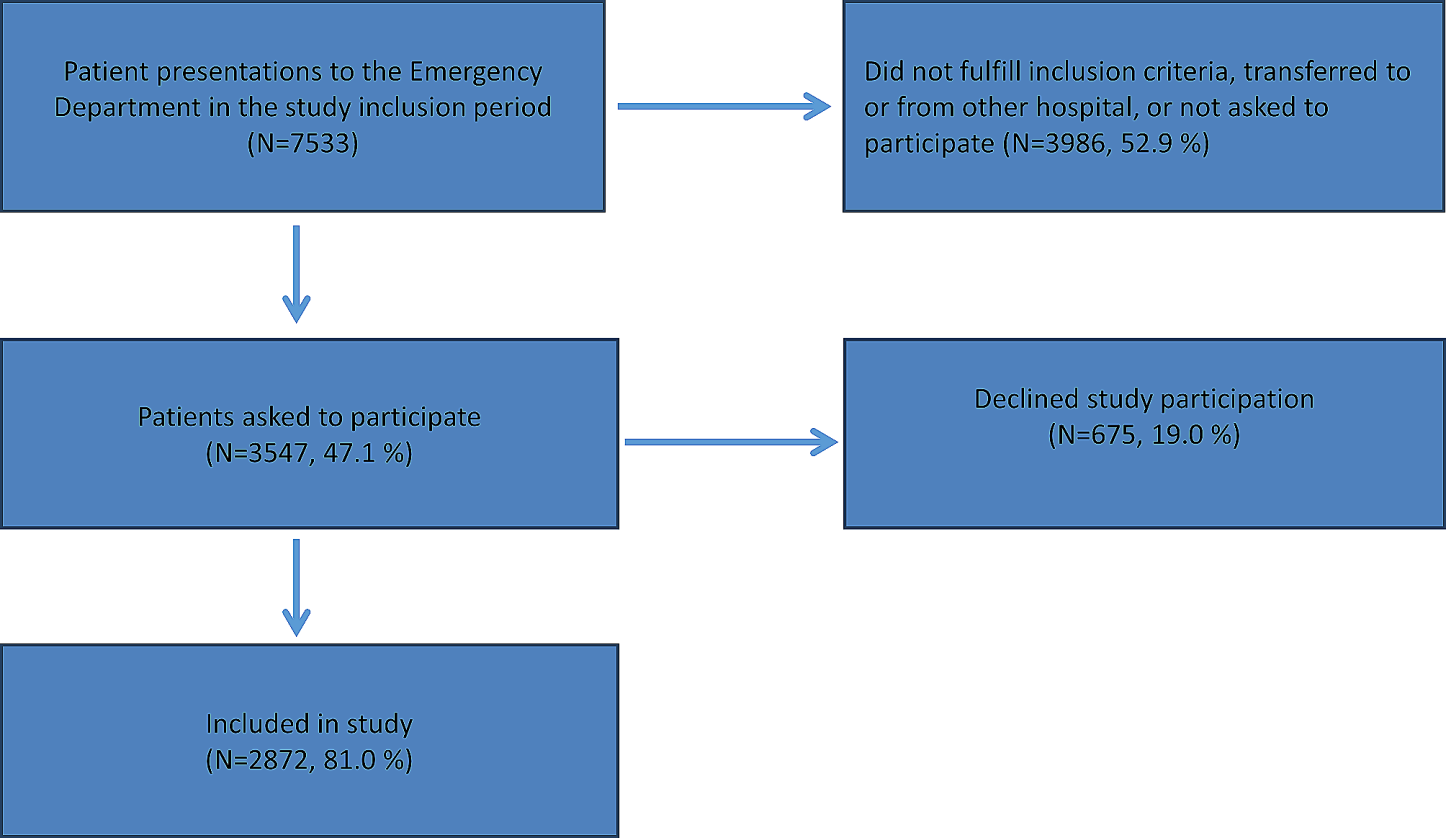
Emergency Department presentations and patient inclusion Legend: Flowchart of patient inclusion at Lovisenberg Diaconal Hospital, conducted from November 2016 to December 2017. Patients were included at all hours of the day.

We utilized data from a prospective cross-sectional study conducted in 2017 at Lovisenberg Diaconal Hospital in Oslo, Norway, involving 2872 patients aged 18 years or older acutely admitted to the Emergency Department [23]. The hospital serves as the local Internal Medicine-center for a catchment area of approximately 180 000 people, consisting of an Intensive Care Unit, a short-term observation unit at the ED, and departments of Cardiology, Geriatric Medicine, Infectious Diseases, Pulmonology, Gastroenterology, and Hematology. Inclusion and exclusion criteria, sample characteristics, alcohol consumption patterns and prevalence rates for psychoactive medication and illicit drug use are available in previous publications [23–25]. The total number of Emergency Department presentations and subsequent inclusion and exclusion rates are summarized in Fig. 1.

### Measures of psychoactive medication use

Psychoactive medicines were detected via analysis of whole blood using liquid chromatography– tandem mass spectrometry [26]. We defined the use of psychoactive medication as the detection of either a benzodiazepine, z-hypnotic, or opioid. We then categorized all positive samples as either “no medication detected”, “single psychoactive medicine detected” or “two or more psychoactive medicines detected”. Methodological details regarding the applied laboratory methods, including lower detection limits, have been presented previously [23].

### Measures of alcohol consumption

Patients self-reported their alcohol consumption through the Alcohol Use Disorder Identification Test 4 (AUDIT-4), with a score ranging from 0 to 16 points [27]. Alcohol consumption was classified as either low-risk drinking or abstinence (0 to 3 points), alcohol use in excess of low-risk guidelines (4 to 6 points), hazardous drinking (7 to 8 points), or risky alcohol use and possible alcohol dependence (9 to 16 points). The above AUDIT-4 categories are consistent with previous publications analyzing data from this population [24], as specific cut-off scores for each degree of alcohol consumption may vary somewhat across different studies [28],

### Length-of-stay and admission frequency

Metrics related to the hospital stay were extracted from the electronic patient journal system. We measured length-of-stay as the total amount of days and hours from arrival at the ED until discharge from any medical ward, including the short-term observation unit. Furthermore, we calculated yearly admission frequency by retrospectively tallying the total number of admissions to our study site the preceding five years. Any registered admission to the ED was included; however, data regarding admissions to hospitals other than the study site were unavailable.

In Norway, out-of-hours emergency wards staffed by general practitioners offer immediate medical care [29] and function as gatekeepers in emergency admissions [30]. As access to specialist healthcare is referral-based, and patients cannot present to the ED without a prior pre-hospital assessment [31], an admission to the ED generally indicates more serious symptoms and conditions. Consequently, we did not set a minimum temporal limit for length-of-stay for inclusion in our analysis, and defined prior admissions as any instance of registered arrival and discharge, including directly from the ED.

### Discharge diagnoses

Primary and secondary diagnoses upon discharge, as defined through the International Classification of Diseases, Tenth Revision [32] [ICD-10], were utilized to classify groups with likely concurrent associations with both study outcomes and exposure variables. A significant percentage of patients with malignant disease satisfy criteria for a psychiatric diagnosis, with high rates of benzodiazepine and z-hypnotic use [33], while opioids are commonly prescribed for managing pain [34]. Cancer patients are also more frequent users of health care services compared to other patient populations [35]. Furthermore, substance use disorders and intoxications are associated with both self-discharge against medical advice, as well as readmission rate and hospitalization length [36, 37]. We therefore identified patients with cancer and metastatic disease (chapter C), substance use disorders (chapter F), and intoxications (chapter T), according to their ICD-10 diagnoses.

### Illicit substances

As data was available [23], and the use of illicit substances may be associated with admission rates [38], we also identified patients whom were positive for at least one illicit substance (tetrahydrocannabinol (THC), amphetamines and methamphetamines, cocaine, methylenedioxy-methylamphetamine (MDMA) and heroin) for use in our analysis.

### Primary outcomes and statistical analysis

Our primary outcome measures were excess length-of-stay and admission frequency, defined as a hospitalization length or yearly admission rate greater than the sample median for the corresponding measurement. As the outcome measures were non-normally distributed, we first assessed median values with interquartile ranges for the entire sample, followed by degrees of psychoactive medication use (none detected, single medicine detected, or two or more medicines detected) and AUDIT-4 score categories (4 to 6, 0 to 3, 7 to 8 and 9 to 16), before examining illicit drug use (none detected versus one or more detected), gender (male/female), age (18 to 64 versus 65 years and older), malignant disease (yes/no), substance use disorder (yes/no) and intoxications (yes/no). In order to obtain median values for both outcome measurements after excluding positive value outliers, we identified values for length-of-stay and admission frequency exceeding 1.5 times the interquartile range above the 75-percentile. Unadjusted within-group comparisons of median length-of-stay and admission frequency across the above measurements were then performed using the independent-samples median test. Employing cross tables and *X*^*2*^-statistics with associated *p*-values, we further calculated the unadjusted distribution above and below the definition for each primary outcome across degrees of psychoactive medication use and AUDIT-4 scores, as well as our co-variates.

We performed separate logistic regression analyses for excess length-of-stay and hospitalization frequency, calculating adjusted estimates for any association between our primary outcomes and the detection of psychoactive medication and degree of self-reported alcohol consumption. We adjusted for age as a continuous variable, gender (male/female), illicit substance use (yes/no), substance use disorders (yes/no), intoxications (yes/no) and malignant disease (yes/no). As alcohol consumption and psychoactive medication use may be interrelated, both of our exposure variables were always included in the analysis. The detection of either a single or two or more psychoactive medicines was compared to our reference category, defined as “no medication detected”. An AUDIT-4 score of 4 to 6 was set as the reference category when examining AUDIT-4 scores of 0 to 3, 7 to 8 and 9 to 16, as patients abstaining from alcohol in a hospital population may do so due to disease burden [39].

Although we judged the definition for excess length-of-stay as appropriate when comparing our sample median to the median value for all patients admitted to our study site (per data from PA Holman, Chief of Analytics, Lovisenberg Diaconal Hospital, May 2021), no such comparison was available for yearly admission frequency. In order to test the strength of any associations, we therefore supplemented our initial analysis by performing the logistic regression after two incremental increases in the value defining our primary outcomes. In place of the sample median, we thus defined excess length-of-stay as longer than 4.0 and 5.0 days, and excess admission frequency as more than 1.0 and 1.5 admissions per year, respectively. Additionally, excluding the sample outliers yielded new, lower median values for both dependent variables. These were therefore also employed as the lower limit for excess length-of-stay and admission frequency in our logistics regression, without including outliers in the analysis.

Our estimates are expressed as odds-ratios (ORs) with 95% confidence intervals. A *p*-value less than 0.05 indicates significance. Any case with a missing variable in either the descriptive analysis or logistic regression was excluded. All data handling and statistical analysis was performed in accordance with relevant guidelines and regulations. Data was analyzed using IBM SPSS 25.0 (Armonk, NY).

## Results

### Sample size

Among the 2872 patients in our sample, post-discharge data regarding length-of-stay was available for 2736 and for admission frequency 2657 - the number of complete datasets after the exclusion of missing or incomplete cases is presented for each individual analysis. Sample characteristics have been detailed previously [23].

### Median length-of-stay across co-variates, psychoactive medication use and alcohol consumption

Median length-of-stay for the entire sample was 3.0 days. In the unadjusted within-group comparison (Table 1), median length-of-stay differed within age group (≥ 65 years, 4.0 days; 18 to 64 years, 2.0 days; *p* < 0.001), low and high AUDIT-4 scores (4 to 6, 2.0 days, reference; 0 to 3, 3.0 days; *p* < 0.001; 9 to 16, 3.0 days; *p* = 0.049), psychoactive medicine use (none detected, 2.0 days, reference; one detected, 3.0 days; *p* < 0.001; two or more detected, 4.0 days; *p* = 0.007), malignant disease status (present, 4.0 days; absent, 2.0 days; *p* < 0.001), illicit drug use (none detected, 3.0 days; one or more detected, 2.0 days; *p* = 0.008) and if diagnosed as an intoxication (yes, 0.63 days; no, 3.0 days; *p* = 0.003).

**Table 1:** Medians with interquartile ranges for length-of-stay and admission frequency across psychoactive medication, alcohol consumption and co-variates

	Length-of-stay (days)		Admission frequency (admissions/year)	
	Median	Interquartile range	Median	Interquartile range
***Entire population***	3.00 *(n = 2736)*	4.50	0.20 *(n = 2657)*	0.40
***Gender***				
Male	3.00 *(n = 1425)*	4.46	0.20 *(n = 1401)*	0.40
Female	2.00 *(n = 1293)*	4.63	0.20 *(n = 1253)*	0.40
***Age (yr)***				
18 to 64	2.00 *(n = 1642)*	3.75	0.00 *(n = 1579)*	0.20
65 and older	4.00* *(n = 1070)*	4.00	0.40* *(n = 1078)*	0.80
***AUDIT-4***				
0–3	3.00* *(n = 1483)*	4.33	0.20 *(n = 1456)*	0.60
4–6	2.00 *(n = 731)*	3.71	0.00 *(n = 707)*	0.20
7–8	2.00 *(n = 189)*	3.75	0.00 *(n = 182)*	0.20
9–16	3.00† *(n = 201)*	4.56	0.20 *(n = 197)*	0.60
***Psychoactive medication***				
None detected	2.00 *(n = 1945)*	3.63	0.00 *(n = 1881)*	0.40
Single detected	3.00* *(n = 465)*	4.33	0.20* *(n = 457)*	0.80
Two or more detected	4.00† *(n = 326)*	4.96	0.40* *(n = 319)*	1.20
***Malignant disease***				
No	2.00 *(n = 2510)*	4.58	0.20 *(n = 2430)*	0.40
Yes	4.00* *(n = 220)*	5.00	0.40* *(n = 218)*	0.80
***Substance use disorder***				
No	2.12 *(n = 2591)*	4.58	0.20 *(n = 2512)*	0.40
Yes	3.00 *(n = 139)*	4.25	0.40* *(n = 136)*	1.15
***Intoxications***				
No	3.00 *(n = 2674)*	4.50	0.20 *(n = 2598)*	0.40
Yes	0.63† *(n = 56)*	1.77	0.20 *(n = 50)*	0.60
***Illicit drugs***				
None detected	3.00 *(n = 2332)*	4.50	0.20 *(n = 2262)*	0.40
One or more detected	2.00† *(n = 157)*	4.27	0.20* *(n = 153)*	0.80

Footnotes: * *p* < 0.001 † *p* < 0.05

Legend: Medians and interquartile ranges for length-of-stay and admission frequency across gender, age, degrees of psychoactive medication use, AUDIT-4-categories, presence of malignant disease and substance use disorder, admittance due to intoxication and detection of illicit drugs. Differences in median values within co-variates were examined using the independent samples median test with associated *p*-values

**Fig. 2:**
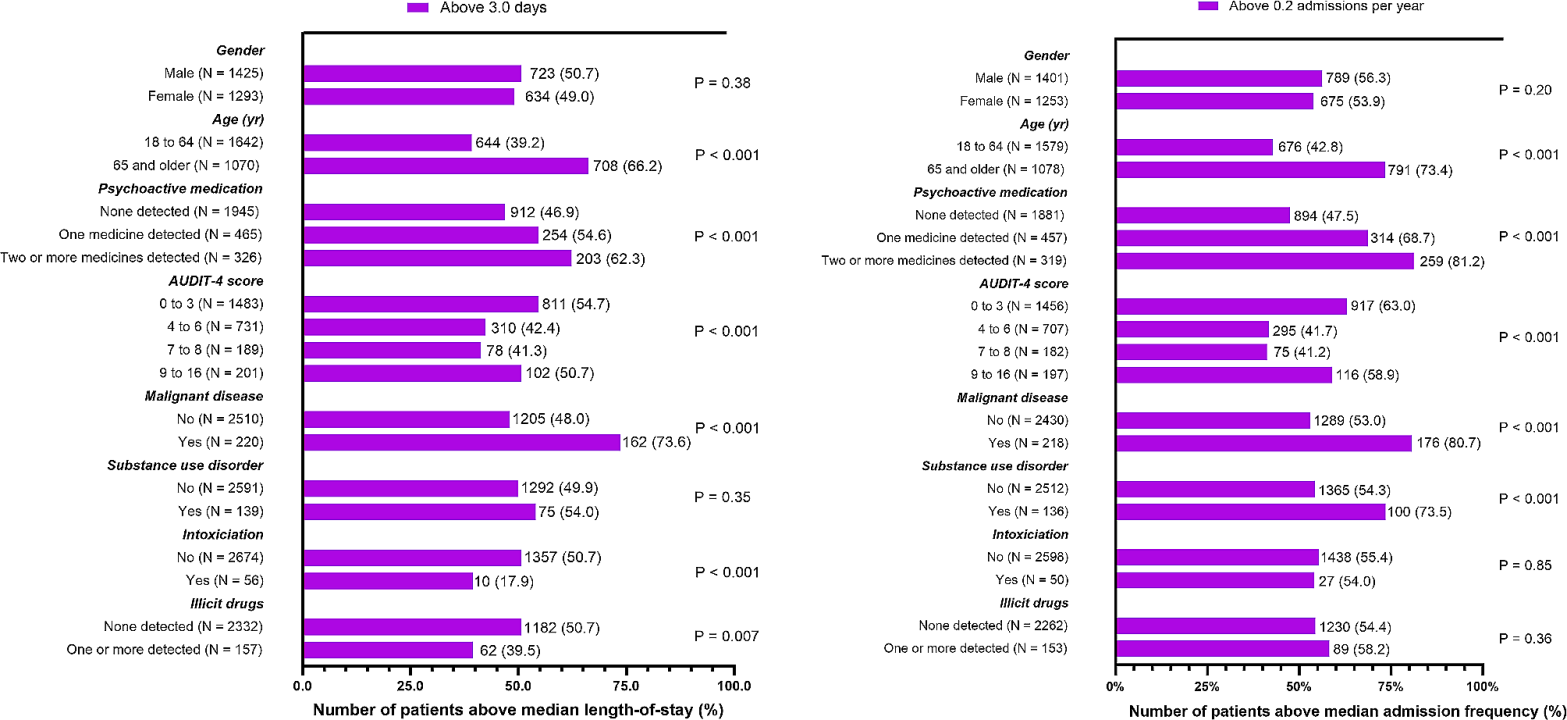
(**A**) Percentage of patients above median length-of-stay across psychoactive medication use, alcohol consumption and co-variates. (**B**) Percentage of patients above median admission frequency across psychoactive medication use, alcohol consumption and co-variates Legend: Within-group distribution of the number of patients above median length-of-stay and admission frequency across gender, age, degrees of psychoactive medication use, AUDIT-4 categories, presence of malignant disease and substance use disorder, admittance due to intoxication and detection of illicit drugs. Significant differences in the percentage of patients defined as having excess length-of-stay or admission frequency when employing the sample median as the lower limit were assessed using *X*^*2*^-statistics with associated *p*-values for each co-variate

Similarly, the percentage of patients above the sample median (Fig. 2A) differed among those aged 65 years and older (66.2%; *p* < 0.001), among AUDIT-4-scores (0 to 3, 54.6%; 4 to 6, 42.4%; 7 to 8, 41.3%; 9 to 16, 50.7%; *p* < 0.001), among degrees of psychoactive medication use (none detected, 46.9%; one detected, 54.6%; two or more detected, 62.3%; *p* < 0.001), when malignant disease is present (73.6%; *p* < 0.001), when classified as an intoxication (17.9%; *p* < 0.001) and when one or more illicit drugs were detected (39.5%; *p* = 0.007).

### Median admission frequency across co-variates, psychoactive medication use and alcohol consumption

Median admission frequency for the entire sample was 0.2 admissions per year (adm/year). As with length-of-stay, there were several differences in the unadjusted within-group comparison (Table 1). Median admission frequency differed within age (≥ 65 years, 0.4 adm/year; 18 to 64, 0.0 adm/year; *p* < 0.001), malignant disease status (present, 0.40 adm/year; absent, 0.20 adm/year; *p* < 0.001), substance use disorder status (present, 0.40 adm/year; absent, 0.20 adm/year; *p* < 0.001) AUDIT-4 scores (4 to 6, 0.0 adm/year, reference; 0 to 3, 0.20 adm/year; *p* < 0.001; 9 to 16, 0.20 adm/year; *p* < 0.001) and psychoactive medication use (none detected, 0.0 adm/year, reference; one detected, 0.20 adm/year; *p* < 0.001; two or more detected, 0.40 adm/year; *p* < 0.001).

There were differences in the percentage of patients above the sample median (Fig. 2B) within age (≥ 65 years, 73.4%; *p* < 0.001), AUDIT-4 scores (0 to 3, 63.0%; 4 to 6, 41.7%; 7 to 8, 41.2%; 9 to 16, 58.9%; *p* < 0.001), psychoactive medication use (none detected, 47.5%; one detected, 68.7%; two or more detected, 81.2%; *p* < 0.001), malignant disease status (present, 73.5%; absent, 54.3%; *p* < 0.001) and substance use disorder status (present, 73.5%; absent, 54.3%; *p* < 0.001).

### Adjusted estimates for excess length-of-stay across psychoactive medication use and alcohol consumption

**Fig. 3:**
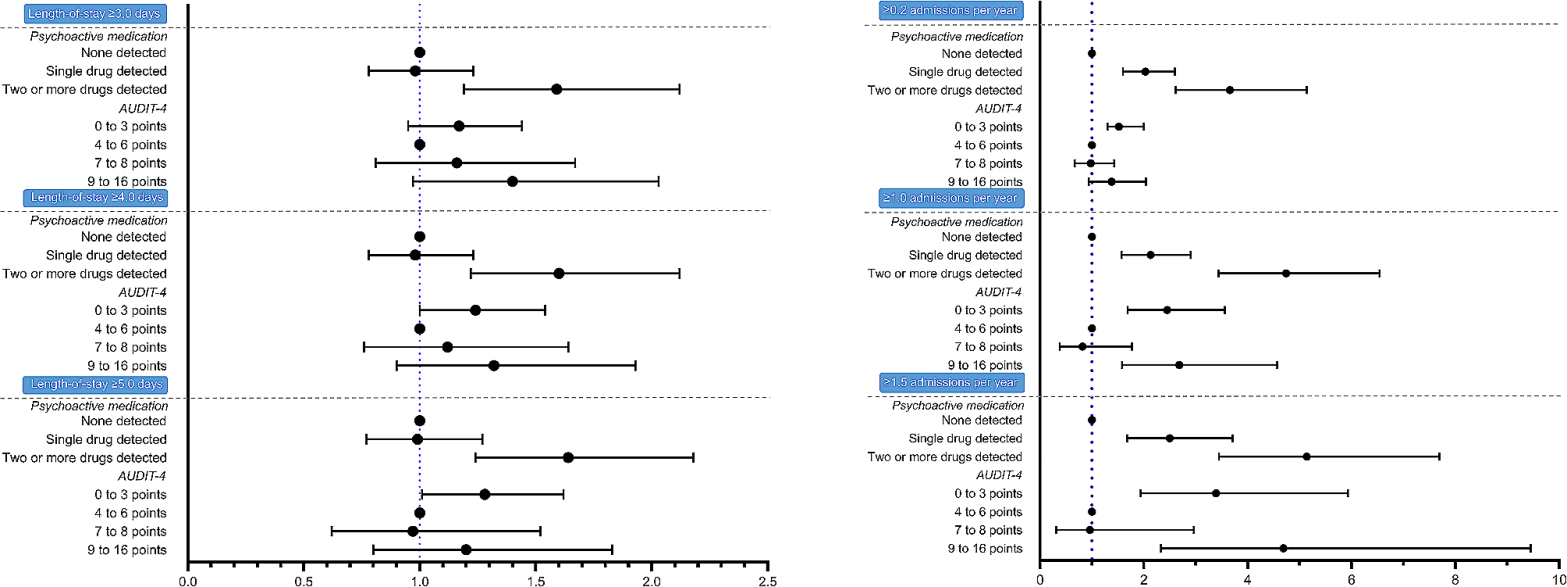
(**A**) Associations between psychoactive medication use, alcohol consumption and excess length-of-stay (*N* = 2353). (**B**) Associations between psychoactive medication use, alcohol consumption and increased admission frequency (*N* = 2301) Legend: Odds-ratios with 95% confidence intervals showing the association between degrees of psychoactive medication use and AUDIT-4-categories, and a length-of-stay above 3, 4 or 5 days, and more than 0.2, 1.0 and 1.5 admissions per year, respectively. Adjusted for age, gender, detection of any illicit drugs, admittance due to intoxication and presence or absence of malignant disease or substance use disorder. Alcohol consumption is adjusted for when examining psychoactive medication use, and vice versa for psychoactive medication use when examining AUDIT-4-scores. The detection of one or two or more psychoactive medicines is compared to no medication detected, and AUDIT-4 scores of 0 to 3, 7 to 8 and 9 to 16 are compared to 4 to 6 points

In our adjusted analysis, with the primary outcome defined as length-of-stay exceeding the sample median of 3.0 days, and psychoactive medication use as an independent categorical variable (Fig. 3A), the detection of two or more psychoactive medicines was associated with excess length-of-stay (odds ratio, 1.59; 95% confidence interval [CI], 1.19 to 2.12; *p* = 0.002), compared to no medication detected. Furthermore, this association persisted when defining excess length-of-stay as longer than 4.0 (odds ratio, 1.60; 95% CI, 1.22 to 2.12; *p* < 0.001) and 5.0 days (odds ratio, 1.64; 95% CI, 1.24 to 2.18; *p* < 0.001). However, the detection of a single psychoactive medicine was not associated with excess length-of-stay whether defined as > 3.0 (odds ratio, 0.98; 95% CI, 0.78 to 1.23; *p* = 0.86), > 4.0 (odds ratio, 0.98; 95% CI, 0.78 to 1.23; *p* = 0.85) or > 5.0 days (odds ratio, 0.99; 95% CI, 0.77 to 1.27; *p* = 0.92).

In the same model, with AUDIT-4-scores as the independent categorical variable and utilizing 3.0 days as the lower boundary, we found no association between excess length-of-stay and scores of 0 to 3 (odds ratio, 1.17; 95% CI, 0.95 to 1.44; *p* = 0.14), and 9 to 16 (odds ratio, 1.40; 95% CI, 0.97 to 2.03; *p* = 0.07), compared to 4 to 6. Effects sizes were further attenuated in scores of 9 to 16 when employing 4.0 (odds ratio, 1.32; 95% CI, 0.90 to 1.93; *p* = 0.15) and 5.0 days (odds ratio, 1.20; 95% CI, 0.80 to 1.83; *p* = 0.39), and conversely, accentuated in scores of 0 to 3 at 4.0 (odds ratio, 1.24; 95% CI, 1.00 to 1.54; *p* = 0.06) and 5.0 days (odds ratio, 1.25; 95% CI, 1.01 to 1.62; *p* = 0.04).

### Adjusted estimates for increased admission frequency across psychoactive medication use and alcohol consumption

Psychoactive medication use was also associated with admission frequency in the adjusted model (Fig. 3B). When defining our primary outcome as exceeding the sample median (0.2 adm/year), the detection of a single psychoactive medicine was associated with increased admission frequency (odds ratio, 2.03; 95% CI, 1.60 to 2.60; *P* < 0.005), compared to none detected. As with length-of-stay, this association persisted when defined as 1.0 (odds ratio, 2.13; 95% CI, 1.57 to 2.90; *p* > 0.005) and 1.5 admissions per year (odds ratio, 2.50; 95% CI 1.68 to 3.71; *p* < 0.005). Additionally, a more pronounced association with increased admission frequency was observed when detecting two or more psychoactive medicines, with successive increases in estimate size when defined as more than 0.2 (odds ratio, 3.66; 95% CI, 2.61 to 5.14; *p* < 0.001), 1.0 (odds ratio, 4.74; 95% CI, 3.44 to 6.54; *p* < 0.001) and 1.5 (odds ratio, 5.14; 95% CI, 3.45 to 7.69; *p* < 0.001) admissions per year.

This association was also present, with a similar increase in effect size, for AUDIT-4 scores of 0 to 3 compared to 4 to 6 when employing > 0.2 (odds ratio, 1.52; 95%CI, 1.30 to 2.00; *p* < 0.005), > 1.0 (odds ratio, 2.45; 95% CI, 1.69 to 3.56; *p* < 0.005) and > 1.5 (odds ratio, 3.39; 95% CI, 1.94 to 5.93; *p* < 0.005) admissions per year. When defined as more than 0.2 admission per year, increased admission frequency was not associated with AUDIT-4 scores of 9 to 16 compared to 4 to 6 (odds ratio, 1.39; 95% CI, 0.94 to 2.04; *p* = 0.10). However, there was an association with more than 1.0 (odds ratio, 2.68; 95% CI, 1.58 to 4.57; *p* < 0.005) and 1.5 (odds ratio, 4.69; 95% CI, 2.33 to 9.45; *p* < 0.005) admissions per year.

### Adjusted estimates for excess length-of-stay and increased admission frequency after outlier exclusion

The median for both dependent variables after excluding positive value outliers was 2.0 days for length-of-stay and 0.0 admissions per year for admission frequency. Adjusted estimates for excess length-of-stay and increased admission frequency using the above median values as the definition were similar to our main analysis (Supplementary Appendix, Tables A1-A2).

## Discussion

In our analysis involving acutely admitted Internal Medicine patients, using psychoactive medication was associated with both excess length-of-stay and increased admission frequency, which was observed for the latter when detecting even a single medicine. The adjusted estimates largely persisted across changes in our outcome definitions. While a non-significant trend toward both excess length-of-stay and hospitalization rate was observed for both very low as well as harmful alcohol consumption, associations were established for more than 1 yearly admission on average, and not for prolonged hospital stays. More attention has been given to the role of psychoactive medication in adverse health outcomes in recent years. A 2017 review of available epidemiological and experimental research indicated a causal relationship between benzodiazepine- and z-hypnotic use and falls, fractures and motor vehicle accidents [40]. Associations have also been found between psychoactive medication use and cognitive impairment [41], as well as adverse respiratory events among older patients with chronic obstructive pulmonary disease [42].

The observational nature of our study limits any causal inferences; however, several mechanisms may have contributed to our results. Previous studies have emphasized the elderly as disproportionally represented among acutely hospitalized Internal Medicine patients [43], whilst simultaneously having higher rates of psychoactive medicine use [20, 44] and a general increase in rates of polypharmacy [45]. However, any patient above the age of 18 able to consent was eligible for inclusion in our study. Consequently, in addition to adjusting for age, our analysis also accounted for intoxications, illicit drug use and substance use disorders, all of which also affect or may be more prevalent in younger individuals [46, 47]. Pertinent considerations among elderly patients nevertheless include changes in the pharmacodynamics and pharmacokinetics of psychoactive medication as a result of senescence, leading to decreased half-life and increased susceptibility to adverse effects [48]. This is further compounded by diversions from prescription guidelines [49], such as not discontinuing long-acting benzodiazepines [50]. A registry-based study from 2012 found inappropriate benzodiazepine use among 12.3% of over 57 000 community dwelling elderly in Norway, based on dosage and duration recommendations [49]. Other studies have shown similar rates [51]. Disconcertingly, benzodiazepine misuse appears to be more common among younger adults [18], while concurrent opioid and either benzodiazepine or z-drug use has been associated with adverse events even when adjusted for age and co-morbidity [52].

Hospital length-of-stay and admission frequency are commonly utilized outcomes when measuring the efficacy of various interventions [5, 53, 54], and as indicators for disease burden, quality of care and medical expenditures [55]. Prolonged hospitalization increases complication risk, such as nosocomial infections, and is costly [56]. Whether the adverse effects of psychoactive medication found in population-based studies are as prominent in hospitalized patients is unclear. However our results raise the question of whether psychoactive medication may prolong hospitalization and increase admission rates, coincident with established risk factors such as age and disease burden. Vigilance regarding inappropriate prescribingshould remain an important tenet among health care providers [57]. Specific interventions in order to reduce the prescription of certain drug classes have already been proven effective in randomized trials [58, 59], where further experimental research examining the effect of drug tapering or discontinuation may shed further light on potential causal mechanisms. Finally, clinicians are encouraged to contextualize harmful alcohol and psychoactive medication use along socioeconomic dimensions, both as risk factors [60] and measures of overall health and disease burden.

Very low and high-risk alcohol high-risk AUDIT-4 scores were associated with hospitalization rates in excess of once yearly. The negative health effects of harmful alcohol consumption are well-established [61], including deleterious effects on existing medical conditions [62] and adverse interactions with concurrent medication, particularly among older adults [63]. When considering low AUDIT-4 scores, alcohol abstainers may in fact do so due to disease severity [39]. Finally, higher rates of premature discharge against medical advice among patients with alcohol use disorders [64] may have confounded our analysis regarding length-of-stay in this group,

While our results appear to be independent of factors associated with both psychoactive medication use and our outcomes, certain limitations are present. The use of psychoactive medication may be a reflection of psychiatric symptoms related to disease burden [65], functioning as an indicator rather than a causal agent. Our sample was not adequately powered in order to examine other disease subgroups where hospitalization may be frequent, such as heart failure [66]. Similarly, assessments of patients with chronic obstructive pulmonary disease (COPD) may be difficult to interpret when identified via ICD-10-codes, which do not specify disease severity and thus cause classification inaccuracy [67]. Other clinically relevant populations, such as patients with cognitive illness and accompanying vulnerability to further decline when using psychoactive medication, may also have been unable to consent and were therefore not included in our sample [57]. Nevertheless, as our study appears to be the first to employ blood sample analysis and questionnaire data in order to examine length-of-stay and hospitalization rate in relation to psychoactive drugs and alcohol, both sample size, inclusion rate and the robustness of our estimates appear to support the validity of our results.

## Conclusion

In this population of Internal Medicine patients, the use of psychoactive medication was associated with both excess length-of-stay and increased admission frequency, with alcohol consumption displaying trends towards both among very low or high-risk drinkers. Whether this represents a causal mechanism, or a reflection of underlying disease burden, should be explored via intervention-based studies. Clinicians must be aware of the potential deleterious effects of psychoactive medicines, particularly among elderly patients.

### Electronic supplementary material

Below is the link to the electronic supplementary material.

Supplementary Material 1

## Data Availability

The datasets generated and/or analyzed during the current study are not publicly available due to an institutional agreement, but anonymized data is available from the corresponding author upon reasonable request.
