# Supplementary material for: Is alcohol and psychoactive medication use associated with excess hospital length-of-stay and admission frequency? A cross-sectional, observational study
Source: BMC Emerg Med. 2024 Apr 16;24:63. doi: 10.1186/s12873-024-00979-y (PMC11020419; doi:10.1186/s12873-024-00979-y)
Supplement: Supplementary file 1 — Supplementary Material 1 [file 12873_2024_979_MOESM1_ESM.docx]

| ***Table A1: Adjusted odds ratios (AOR) for the association between psychoactive medication use, alcohol consumption and excess length-of-stay (N=2292)* after outlier exclusion^†^*** | | | | | | |
| --- | --- | --- | --- | --- | --- | --- |
|  |  |  |  |  |  |  |
|  | **≥ 2.0 days** |  | **≥3.0 days** |  | **≥4.0 days** |  |
|  | AOR (95 % CI) | P-value | AOR (95 % CI) | P-value | AOR (95 % CI) | P-value |
| No psychoactive medication detected | Ref |  | Ref |  | Ref |  |
| One psychoactive medicinal drug detected | 0.97 (0.78 to 1.23) | P = 0.80 | 0.94 (0.75 to 1.19) | P = 0.61 | 0.94 (0.74 to 1.10) | P = 0.61 |
| Two or more psychoactive medicinal drugs detected | 1.52 (1.14 to 2.04) | P = 0.004 | 1.49 (1.12 to 1.99) | P = 0.006 | 1.53 (1.16 to 2.03) | P = 0.003 |
| AUDIT 0 to 3 | 1.14 (0.93 to 1.40) |  | 1.15 (0.94 to 1.42) | P = 0.18 | 1.21 (0.97 to 1.51) | P = 0.09 |
| AUDIT 4 to 6 | Ref |  | Ref |  | Ref |  |
| AUDIT 7 to 8 | 1.13 (0.79 to 1.63) | P = 0.49 | 1.17 (0.82 to 1.68) | P = 0.39 | 1.12 (0.75 to 1.66) | P = 0.58 |
| AUDIT 9 to 16 | 1.35 (0.94 to 1.94) | P = 0.11 | 1.39 (0.96 to 2.00) | P = 0.08 | 1.36 (0.91 to 1.96) | P = 0.14 |

* Adjusted for age, gender, AUDIT-4-score, illicit drugs, substance use disorders, intoxications, and malignant disease

† Outliers calculated as having a length-of-stay longer than 12.0 days.

| ***Table A2: Adjusted odds ratios (AOR) for the association between psychoactive medication use, alcohol consumption and increased admission frequency (N=2031)* after outlier exclusion^†^*** | | | | | | |
| --- | --- | --- | --- | --- | --- | --- |
|  |  |  |  |  |  |  |
|  | **> 0.0 adm/year** |  | **≥ 0.2 adm/year** |  | **≥ 1.0 adm/year** |  |
|  | AOR (95 % CI) | P-value | AOR (95 % CI) | P-value | AOR (95 % CI) | P-value |
| No psychoactive medication detected | Ref |  | Ref |  | Ref |  |
| One psychoactive medicinal drug detected | 1.84 (1.43 to 2.37) | P < 0.001 | 1.84 (1.43 to 2.37) | P < 0.001 | 1.74 (0.90 to 3.37) | P = 0.10 |
| Two or more psychoactive medicinal drugs detected | 2.68 (1.87 to 3.84) | P < 0.001 | 2.68 (1.87 to 3.84) | P < 0.001 | 2.94 (1.46 to 5.95) | P = 0.003 |
| AUDIT 0 to 3 | 1.47 (1.18 to 1.84) | P < 0.001 | 1.47 (1.18 to 1.84) | P < 0.001 | 2.51 (1.15 to 5.48) | P = 0.02 |
| AUDIT 4 to 6 | Ref |  | Ref |  | Ref |  |
| AUDIT 7 to 8 | 0.97 (0.66 to 1.43) | P = 0.88 | 0.97 (0.66 to 1.43) | P = 0.88 | 0.89 (0.19 to 4.30) | P = 0.89 |
| AUDIT 9 to 16 | 1.09 (0.72 to 1.65) | P = 0.68 | 1.09 (0.82 to 1.65) | P = 0.68 | 0.32 (0.04 to 2.76) | P = 0.30 |

* Adjusted for age, gender, AUDIT-4-score, illicit drugs, substance use disorders, intoxications, and malignant disease

† Outliers calculated as having an admission frequency above 1.0 admissions per year.
